# Enabling witnesses to actively explore faces and reinstate study-test pose during a lineup increases discriminability

**DOI:** 10.1073/pnas.2301845120

**Published:** 2023-10-02

**Authors:** Marlene Meyer, Melissa F. Colloff, Tia C. Bennett, Edward Hirata, Amelia Kohl, Laura M. Stevens, Harriet M. J. Smith, Tobias Staudigl, Heather D. Flowe

**Affiliations:** ^a^School of Psychology, University of Birmingham, Edgbaston B15 2TT, United Kingdom; ^b^Department of Sport and Health Sciences, Technical University of Munich, Munich 80333, Germany; ^c^School of Psychology, University of Bath, North East Somerset, South West BA2 7AY, United Kingdom; ^d^School of Psychology, Nottingham Trent University, Nottingham NG1 4FQ, United Kingdom; ^e^Department of Psychology, Ludwig-Maximilians-Universität München, Munich 80539, Germany

**Keywords:** eyewitness identification, interactive lineup, diagnostic feature detection theory, encoding specificity

## Abstract

Accurate witness identification is a cornerstone of police inquiries and national security investigations. However, witnesses can make errors. We experimentally tested whether an interactive lineup, a recently introduced procedure that enables witnesses to dynamically view and explore faces from different angles, improves the rate at which witnesses identify guilty over innocent suspects compared to procedures traditionally used by law enforcement. Participants encoded 12 target faces, either from the front or in profile view, and then attempted to identify the targets from 12 lineups, half of which were target present and the other half target absent. Participants were randomly assigned to a lineup condition: simultaneous interactive, simultaneous photo, or sequential video. In the front-encoding and profile-encoding conditions, Receiver Operating Characteristics analysis indicated that discriminability was higher in interactive compared to both photo and video lineups, demonstrating the benefit of actively exploring the lineup members’ faces. Signal-detection modeling suggested interactive lineups increase discriminability because they afford the witness the opportunity to view more diagnostic features such that the nondiagnostic features play a proportionally lesser role. These findings suggest that eyewitness errors can be reduced using interactive lineups because they create retrieval conditions that enable witnesses to actively explore faces and more effectively sample features.

## Significance

Incorrect lineup identification decisions have devastating consequences on the wrongfully accused and for society when guilty perpetrators remain at large. This paper heeds the call by the National Academy of Sciences to utilize technology to increase correct identifications and minimize incorrect identifications. We found that the simultaneous interactive lineup procedure, which allows witnesses to actively explore the lineup members’ faces, increases discriminability compared to traditionally used video and simultaneous photo lineups. Interactive lineups were beneficial regardless of perpetrator encoding viewpoint (i.e., front or profile). In the real world, eyewitnesses may view a perpetrator from multiple perspectives. The interactive procedure allows witnesses to adjust face viewpoints to match their memory of the crime, which may be beneficial for memory retrieval.

Eyewitness identification is a cornerstone of police inquiries and national security investigations. However, mistaken identification has been implicated in about 70% of U.S. wrongful convictions (1). Failures to identify guilty suspects mean missed opportunities to arrest guilty people, allowing them to commit additional crimes. Therefore, implementing procedures that enhance discriminability (i.e., that maximize correct and minimize incorrect suspect identifications) is an important public policy goal (2).

Nevertheless, the technology used to conduct identifications has not fundamentally changed over the past century. The static 2D photo lineup is the most widely used procedure worldwide (3). A lineup contains the police suspect, who may be guilty or innocent, and several fillers, who physically resemble the suspect and are known to be innocent of the crime. The members are presented head-on, in frontal view. If the lineup is target present (i.e., contains the guilty suspect), the witness could correctly identify the suspect or err by choosing a filler or rejecting the lineup (i.e., choosing no one). If the lineup is target absent (i.e., the guilty suspect is absent), the witness could correctly reject the lineup or err by choosing an innocent suspect or a filler. Witnesses in experiments make mistakes around half the time (4) and in real world cases frequently identify known-innocent suspects (5).

A large body of research dedicated to improving discriminability has accumulated. In laboratory studies, participant witnesses are shown a mock crime and tested under different lineup conditions. Many studies have compared sequential to simultaneous lineups, wherein, respectively, witnesses view the members one at a time versus all together. In research comparing simultaneous to sequential lineups, some studies have found discriminability is higher in simultaneous lineups (e.g., ref. 6), while others have found witnesses’ response bias, or willingness to make a positive identification, is more lenient in simultaneous lineups, resulting in more guilty and innocent suspect identifications (e.g., ref. 7).

Laboratory research has also examined video lineups, which are used in the U.K. and presented sequentially (8), with each member turning their head left and right to show the faces from every angle. Some studies have found discriminability is higher in static photo simultaneous compared to video lineups (9–11), while others have found response bias is stricter in video than photo lineups (e.g., ref. 12) or no differences (13). However, none of the studies systematically controlled the perpetrator’s face angle during encoding. If participants in past research largely encoded the perpetrator’s face in frontal view, any benefit of face angle as a retrieval cue would be masked, since all procedures allow witnesses to see the members in frontal view.

In this study, we heed the National Academy of Sciences call for technology to improve discriminability (14) and investigate whether enabling witnesses to actively explore the members’ faces along the vertical axis from −90° to 90° improves discriminability compared to sequential video and simultaneous photo lineups, the two most widely used procedures worldwide. We experimentally controlled encoding angle.

Discriminability should theoretically be higher in interactive compared to existing procedures since witnesses can engage in *pose reinstatement*, or rotate the lineup faces to match the angle in which the perpetrator was encoded. This is because encoding specificity, or the match between cues at encoding and retrieval, is important for memory retrieval (15, 16). Further, according to diagnostic feature detection theory, discriminability is higher if the witness can detect diagnostic features (i.e., features that match the perpetrator only) and discount nondiagnostic features (i.e., features shared by all lineup members) (2). Therefore, discriminability should be higher if witnesses can evaluate the members’ facial features from the same angle as they as they encoded the perpetrator. Affording witnesses the opportunity to consider a greater number of (diagnostic) facial features across members in the same angle means that nondiagnostic features play a proportionally lesser role. The ability of witnesses to actively explore faces may also be a contributing factor. Active exploration can enhance memory performance (17), perhaps by facilitating the intentional sampling of relevant facial features (18), which again could enhance the ability to perceive predominantly more diagnostic features.

Drawing on the theory and research outlined above, Colloff et al. (19, 20) developed and tested interactive lineups. Discriminability was higher for witnesses who could reinstate pose (ref. 19, Exp. 1). Without prompting, participant witnesses tended to rotate the interactive lineup members’ faces into the same angle as they saw the perpetrator commit the crime, and discriminability was higher for those who interacted more (ref. 19, Exp. 2). This suggests the structure of eyewitness memory includes information about face angle and allowing witnesses to utilise these cues facilitates memory retrieval. In another project that tested almost 10,000 participants, Colloff et al. (20) compared discriminability in sequential photo versus sequential interactive lineups (Exp. 1), and in interactive lineups presented simultaneously versus sequentially (Exp. 2). Discriminability was higher in interactive than photo sequential lineups and was boosted further with the simultaneous presentation of interactive faces. This suggests the ability to actively explore and compare faces from multiple angles allows diagnostic features to carry more weight during memory retrieval, facilitating accuracy.

## Predictions

This study substantially extends previous theory and research by comparing discriminability in simultaneous interactive lineups against sequential video and simultaneous frontal pose static photo lineups.

Diagnostic feature detection theory predicts that discriminability is higher when witnesses can better detect and discount features that are nondiagnostic (2), such as when lineup members are viewed simultaneously versus sequentially. Additionally, encoding specificity predicts higher discriminability when encoding and test cues match. Therefore, for front-encoding, we predicted that discriminability would be higher in simultaneous interactive and simultaneous photo lineups versus sequential video lineups due to simultaneous presentation. No differences in accuracy were predicted for interactive simultaneous versus photo simultaneous lineups, as encoding and test cues match in both procedures. For profile-encoding, we predicted that discriminability would be higher in simultaneous interactive versus sequential video lineups because of the benefit of showing members together, and higher in simultaneous interactive versus simultaneous photo because witnesses can reinstate pose so that encoding and test cues match. For simultaneous photo versus sequential video lineups, we did not make a directional prediction: Accuracy could be higher in simultaneous photo versus sequential video lineups because the members can be compared simultaneously; however, accuracy could be higher in video versus photo lineups owing to pose reinstatement.

## Results

Thirty-one participants in the interactive condition did not interact with any of the faces in the twelve lineups as instructed. We did not anticipate this and therefore did not preregister that we would remove participants who failed to follow instructions. However, by not interacting, the interactive lineup becomes equivalent to the photo lineup, making it difficult to meaningfully compare procedures. Further, discrimination accuracy did not differ for interactors compared to noninteractors, suggesting the participant groups were similarly motivated to make an accurate identification [front encoding: interactors (*n* = 65; *d*  ′ = 1.47) versus noninteractors (*n* = 18, *d*  ′ = 0.80), G = 2.06, *P* = 0.98; profile encoding: interactors (*n* = 78, *d*  ′ = 1.28) versus noninteractors (*n* = 13, *d*  ′ = 1.18), G = 0.98, *P* = 0.83]. An analysis of all participant data (including noninteractors) is in *SI Appendix*. *SI Appendix* *p*rovides ID decision frequencies by confidence level without and with interactors (*SI Appendix*, Tables S2 and S3), and analyses of decision time by experimental condition (Table S1). None of the differences in discriminability can be explained by decision time differences across the lineup conditions.

### Lineup Identification Decisions.

Table 1 shows the total number and proportions of target, filler, and “Not present” (reject) identification (ID) decisions across conditions. Interactive lineups appeared to yield better performance in both the front- and profile-encoding conditions versus simultaneous photo and video lineups.

**Table 1.** Totals and proportions of target, filler, and reject identification decisions for front- and profile-encoding conditions in interactive, photo, and video lineups. Excluding 31 Noninteractors from the interactive condition

		Front-encoding					Profile-encoding				
		Target-present			Target-absent		Target-present			Target-absent	
		Target	Filler	Reject	Filler	Reject	Target	Filler	Reject	Filler	Reject
Interactive	Total	173	102	109	193	191	175	165	128	239	229
	Proportion	0.45	0.27	0.28	0.50	0.50	0.37	0.35	0.28	0.51	0.49
Photo	Total	203	149	140	258	234	139	211	166	308	208
	Proportion	0.41	0.30	0.29	0.52	0.48	0.27	0.40	0.33	0.60	0.40
Video	Total	145	122	117	201	183	108	160	152	248	172
	Proportion	0.38	0.32	0.30	0.52	0.48	0.26	0.38	0.36	0.59	0.41

Note. Total rows contain the frequency of every identification decision collapsed over participants and confidence. Proportion rows are calculated by dividing the number of identification decisions by the number of lineups in a particular condition. For instance, the proportion of front-encoding target identifications in interactive lineups is computed by dividing the number of target identifications in target-present front-encoding interactive lineups by the total amount of target-present front-encoding interactive lineups, 173/(173 + 102 + 109).

### Receiver Operating Characteristics (ROC) Analysis.

**Fig. 1.**
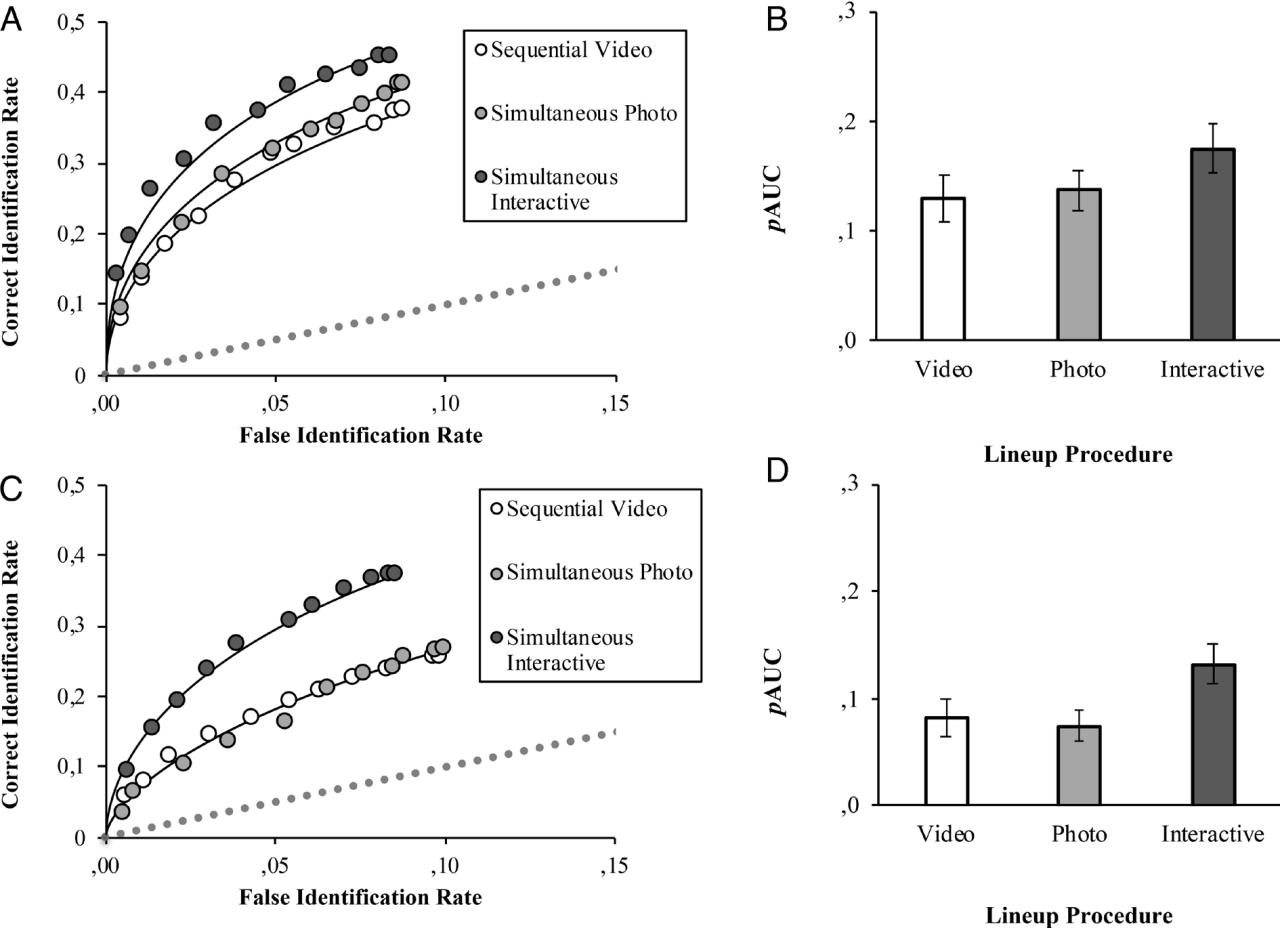
pROC curves and pAUC statistics. Note. *p*ROC curves and *p*AUC statistics for simultaneous interactive, simultaneous static photo and sequential video lineups, separated for front (*A* and *B*) and profile (*C* and *D*) encoding conditions. ROC lines of best fit were plotted from numbers estimated by unequal-variance signal-detection models. Chance-level performance is indicated by dashed lines. For *p*AUC values (*B* and *D*), error lines are 95% CIs.

ROC analysis followed Mickes et al. (6) and the partial Area Under the Curve (*p*AUC) was calculated using statistical package *p*ROC (21). Alpha was set at 0.05, with one-tailed tests for directional hypotheses, and two-tailed tests for nondirectional hypotheses. Fig. 1 displays partial ROC curves with attendant *p*AUC values by lineup condition for front- (specificity = 0.50) and profile-encoding (specificity = 0.49). The relative height of the ROC curves indicates that participants more accurately discriminated between innocent and guilty suspects in interactive versus photo and video lineups regardless of encoding angle.

For front-encoding, the *p*AUC was larger for interactive (0.174) versus simultaneous photo (0.137) lineups, *D* = 2.423, *P* = 0.015 (two-tailed), which was not predicted. In line with diagnostic feature detection, the *p*AUC was greater for interactive versus video (0.129) lineups, *D* = 2.803, *P* = 0.002 (one-tailed); but, contrary to diagnostic feature detection, the *p*AUCs for photo versus video lineups did not differ, *D* = 0.539, *P* = 0.29 (one-tailed).

For profile-encoding, the *p*AUC was larger for interactive (0.131) versus photo (0.075) lineups, *D* = 4.651, *P* < 0.001 (one-tailed), as predicted by encoding specificity. Moreover, the *p*AUC was larger for interactive versus video (0.082) lineups, *D* = 3.957, *P* < 0.001 (one-tailed), as predicted by diagnostic feature detection. The *p*AUCs for video versus photo lineups did not differ, *D* = 0.630, *P* = 0.53 (two-tailed).

### Maximum Likelihood Signal-Detection Model Fits.

Since ROC analysis is an atheoretical measure, we validated our findings with theoretical measures, fitting signal-detection models to the data (2, 22). The 11-point confidence scale was combined into a 3-point scale to decrease the number of model parameters. We combined confidence ratings of 0 to 60% as *c_1_*,70 to 80% as *c_2,_* and 90 to 100% as *c_3_*(see ref. 20). Higher *c* parameter estimates indicate increasingly conservative responding, whereby more memory evidence is required before making an identification. We used the independent observation model with a correlation parameter (23), which assumes that an identification is made when the most familiar face in the lineup exceeds *c_1_*. If no face in the lineup is familiar enough to exceed *c_1_*, the lineup is rejected. Identification confidence is determined by the highest criterion exceeded. The correlation parameter (σ*_b_*) allows the correlation between the suspect and fillers to vary from 0, because faces in a fair lineup match the witness’s description and therefore should be theoretically correlated (23). We conducted the model-fitting separately for the front- and profile-encoding conditions.

To estimate summary discriminability (ability to discriminate innocent from guilty suspects), correlation, and confidence criteria parameters, the model was fit to the target, filler, and reject decisions in the three lineup conditions. The mean and standard deviation of the innocent distribution was set to 0 and 1, by convention. An unequal variance model was used, because σ_target_ was always estimated to be significantly larger than σ_innocent_, as it should have been, as there were multiple target faces at encoding which presumably adds random noise to the process. Model-estimated σ_target_ was constrained to be the same across the three lineup conditions (1 model parameter), because allowing it to differ across conditions never significantly improved the fit. The estimated value of the correlation parameter (σ*_b_*) never significantly differed from 0 (though it theoretically should have); so it plays no role in the following model fits but is considered later.

Discriminability (*d*) values were allowed to vary across the lineup conditions by allowing μ_target_ to vary, so the *full model* had 14 degrees of freedom: 27 [3 lineup conditions × 9 (target, TP and TA filler IDs at 3 levels of confidence)] – 13 parameters [3 lineup conditions × 4 (μ_target_, *c_1_*, *c_2_*, *c_3_*) and σ_target_]. To test if any differences in *d* were statistically significant, we fit a series of reduced models (essentially three pairwise comparisons), constraining *d* to be the same across two conditions and compared the fit of the reduced to the full model (Table 2). For front-encoding the *full model* explained the data well (χ^2^(14) = 12.39, *P* = 0.575). Discriminability was larger in interactive (*d* = 1.32) than photo (*d* = 1.12) lineups, but this difference was not statistically significant (χ^2^(1) = 3.58, *P* = 0.058). Discriminability was significantly larger in interactive compared to video (*d* = 1.00) lineups (χ^2^(1) = 7.48, *P* = 0.006). There was no significant difference in discriminability between photo and video lineups (χ^2^(1) = 1.02, *P* = 0.313). For profile encoding, the *full model* explained the data well (χ^2^(14) = 12.32, *P* = 0.581). Discriminability was significantly larger in interactive (*d* = 1.04) than in photo (*d* = 0.60, χ^2^(1) = 19.02, *P* < 0.001) and video (*d* = 0.60, χ^2^(1) = 16.86, *P* < 0.001) lineups, but did not differ between photo and video lineups (χ^2^(1) = 0.00, *P* = 1.000).

**Table 2.** Fitted models for the interactive, photo, and video lineup d Comparisons for front- and profile-encoding

	Front encoding					Profile encoding				
Model	μ_target_/*d*	σ_target_	*c_1_*	*c_2_*	*c_3_*	μ_target_/*d*	σ_target_	*c_1_*	*c_2_*	*c_3_*
Full									
Interactive	1.32		1.25	1.98	2.42	1.04		1.18	1.83	2.19
Photo	1.12	1.25	1.20	1.82	2.40	0.60	1.17	1.09	1.77	2.34
Video	1.00		1.20	1.90	2.30	0.60		1.13	1.86	2.27
Model-fit	χ^2^(14) = 12.39, *P* = 0.575					χ^2^ (14) = 12.32, *P* = 0.581				
Reduced interactive-photo comparison									
Interactive	1.21		1.23	1.96	2.40	0.81		1.16	1.80	2.15
Photo		1.25	1.21	1.83	2.42		1.18	1.11	1.80	2.37
Video	1.00		1.20	1.90	2.30	0.59		1.13	1.86	2.27
Model-fit	χ^2^(15) = 15.97, *P* = 0.389					χ^2^(15) = 31.34, *P* = 0.008				
Reduced interactive-video comparison									
Interactive	1.16		1.23	1.96	2.39	0.84		1.16	1.80	2.15
Photo	1.12	1.26	1.20	1.82	2.40	0.59	1.17	1.09	1.77	2.34
Video	1.16		1.22	1.92	2.33	0.84		1.15	1.89	2.31
Model-fit	χ^2^(15) = 19.87, *P* = 0.177					χ^2^(15) = 29.18, *P* = 0.015				
Reduced photo-video comparison									
Interactive	1.32		1.25	1.98	2.42	1.04		1.18	1.83	2.19
Photo	1.07	1.25	1.19	1.81	2.39	0.60	1.17	1.09	1.77	2.34
Video			1.21	1.91	2.31			1.13	1.86	2.27
Model-fit	χ^2^(15) = 13.44, *P* = 0.568					χ^2^(15) = 12.32, *P* = 0.655				

Note. In the *full model d* varies across conditions. In the *reduced models*, the two procedures that are compared are restricted to an equal *d*. In both full and reduced models, model-estimated σ_target_ was constrained to be the same over conditions, and *c_1_, c_2_*, and *c_3_* were free to vary. Model-fit rows represent the goodness-of-fit statistic.

The independent observation model that allows for a positive correlation among the memory signals of a lineup effectively operates like diagnostic feature detection theory under certain conditions by reducing the impact of the shared nondiagnostic features and increasing the pAUC. One way to reduce the impact of shared features is by increasing the correlated memory signals between faces (hereafter, the *correlational account*). This could explain why lineups that have correlated memory signals (owing to shared features across faces) yield higher discriminability than showups (a single face, where the concept of correlated memory signals does not apply see ref. 24). Another way to reduce the impact of shared features is to increase the number of diagnostic features available (increasing the total number of facial features considered). In which case, the shared features play a proportionally lesser role, thereby decreasing the correlation but increasing discriminability as the memory strength distributions for innocent and guilty lineup members become further apart (hereafter, the *distributional account*) (25).

To investigate the foregoing, we fit a model allowing μ_target_ (*d,* the distance between the guilty and innocent distributions) and σ_b_ (the correlation) to differ across the lineup conditions. Again, the model-estimated σ_target_ was constrained to be the same across the lineup conditions, and the confidence criteria could vary. We found that for both front- and profile-encoding, the correlation is smallest and *d* is largest in the interactive condition, consistent with the distributional account (Table 3, see *SI Appendix* *f*or model fits).

**Table 3.** Fitted models for the interactive, photo and video lineups estimating correlation (σ_b_) and distributional (μ_target_) differences

Model	μ_target_/*d*	σ_target_	σ_b_	*c_1_*	*c_2_*	*c_3_*
Front encoding						
Interactive	1.31		0.09	1.24	1.99	2.43
Photo	1.04	1.30	0.46	1.04	1.74	2.36
Video	0.92		0.46	1.05	1.83	2.26
Model-fit	χ^2^(11) = 11.26, *P* = 0.422					
Profile encoding		
Interactive	1.03		0.00	1.18	1.83	2.19
Photo	0.49	1.19	0.58	0.82	1.63	2.26
Video	0.58		0.01	1.13	1.86	2.27
Model-fit	χ^2^(15) = 12.32, *P* = 0.655					

Note. σ_target_ was set to be the same over conditions, and *c_1_, c_2_* and *c_3_* were free to vary. Correlation *r* = σ_b_^2^. Model-fit rows represent the goodness-of-fit statistic.

Overall, the signal-detection modeling validates the ROC analysis results, though for front-encoding, increased discriminability for the interactive compared to the photo lineup did not reach statistical significance in the modeling. The discriminability improvement observed for interactive lineups is most parsimoniously explained by the distributional account.

## Discussion

We compared the simultaneous interactive to simultaneous photo and sequential video lineups, two widely used police procedures worldwide. Encoding angle (front or profile) was systematically controlled. For front-encoding, discriminability was significantly higher in simultaneous interactive compared to sequential video lineups and simultaneous photo lineups; but, this latter difference was statistically significant only in the ROC analysis and not the modeling. For profile-encoding, discriminability was higher in simultaneous interactive compared to simultaneous photo and sequential video lineups, which did not differ from each other (see *SI Appendix* *f*or results with noninteractors included). This suggests active exploration and encoding specificity boost discriminability. Further, the modeling results suggest interactive lineups move the memory strength distributions for innocent and guilty lineup members further apart, ostensibly because they increase the number of diagnostic features available.

### Interactive versus Photo and Video Lineups.

For front encoding, for any false ID rate, simultaneous interactive lineups enhanced the correct ID rate of target faces by 35% and 27% compared to sequential video and simultaneous photo lineups, respectively. The ROC analysis found better discriminability for simultaneous interactive than simultaneous photo lineups, though this was not statistically significant in the modeling results (*P* = 0.058). The front-encoding findings suggest that active over passive exploration enhances feature sampling (17), aiding diagnostic feature detection (2). This notion aligns well with studies demonstrating a relationship between visual exploration and memory performance. For example, complex stimuli explored with more eye movements are better remembered than stimuli explored with fewer eye movements (e.g., ref. 26).

For profile-encoding, for any possible false identification rate, interactive lineups enhanced the target correct identification rate by 75% and 60% compared to simultaneous photo and video lineups, respectively. This suggests discriminability is enhanced for simultaneous interactive witnesses because they can actively explore and compare faces in the same pose that they were encoded, thereby increasing the availability and use of proportionally more diagnostic features. Further research is needed to examine the relative contributions of active exploration and pose reinstatement in enhancing discriminability. This work should also incorporate independent measures of participant motivation and engagement to further investigate any potential biases that might be introduced by the exclusion of noninteracting participants from the analysis.

### Photo versus Video.

For both front- and profile-encoding, discriminability did not differ between simultaneous photo and sequential video lineups. For front-encoding, this does not support our prediction from the diagnostic-feature-detection theory (2) that simultaneous presentations (in this case, the photo lineup) enhance discriminability compared to sequential presentations (i.e., the video lineup). Findings are mixed, however. Fitzgerald et al. (3) concluded that video and photo lineups yield comparable discriminability, and no procedure is favorable over the other. However, video lineups are typically sequential, and photo lineups--although they *can* be sequential--are typically simultaneous, and this may affect performance. Seale-Carlisle and Mickes (10) found higher discriminability for simultaneous photo compared to sequential video lineups, and Seale-Carlisle et al. (11) found simultaneous photo and simultaneous video lineups did not differ. Further research considering encoding conditions is needed to clarify this debate.

### Summary.

This study demonstrates how psychology research grounded in strong theory from basic science can be tested and applied to improve forensic science, and particularly eyewitness memory performance. In the real world, it is likely that eyewitnesses will view a perpetrator from multiple perspectives (particularly nonfrontal) (27), and the interactive procedure allows witnesses to adjust lineup face viewpoints to match their memory of the crime, which appears to be particularly beneficial for memory performance. If research continues to evidence an interactive benefit, simultaneous interactive lineups could be adopted by police forces globally to allow for encoding-retrieval matching and increased ability for diagnostic feature comparison.

## Materials and Methods

The methods and analyses were OSF preregistered (https://osf.io/rkafh/).

### Participants.

In all, 550 participants were recruited using Amazon Mechanical Turk (www.mturk.com) and compensated £2.70 for participating. Totally, 75 individuals were excluded based on a failed attention check (*n* = 71) and technical problems during the experiment (*n* = 4). The final sample (N = 475) was 52% female and 47% male (1% preferred not to say), and 73% Caucasian, 14% Black, 7% Asian, and 6% other.

Participants were randomly assigned to one of the six between-subject conditions (front-interactive *n* = 82, profile-interactive *n* = 91, front-photo *n* = 82, profile-photo *n* = 86, front-video *n* = 64, profile-video *n* = 70).

### Design.

A 2 (encoding: front or profile view) × 3 (procedure: interactive, photo, or video) × 2 (target: present or absent) mixed design was used. Encoding and procedure were varied between subjects, while target was varied within subjects. Each participant learned twelve faces at encoding (front view or profile view) and was tested on twelve lineups, of which, six were target-present and six were target-absent. The outcome variables were identification accuracy and decision confidence.

We used ROC analyses because this method has been successfully applied in lineup research (6) and can quantify participants’ ability to discriminate innocent from guilty suspects (28–30). For stable functions, ROC analysis requires large samples. ROC lineup studies typically recruit approximately 500 data points per condition, so for our study we adopted a data collection stopping rule of 6,000 data points (500 participants × 12 conditions = 6,000).

Ethical approval was granted from the Science, Technology, Engineering and Mathematics Ethical Review Committee at the University of Birmingham.

### Materials.

Materials were adapted from Colloff et al. (20).

#### Photographs.

Twelve frontal pose and twelve profile view photographs served as targets (400 × 300 pixels), each showing a face from shoulders up. The targets were male (*n* = 8) and female (*n* = 4), and Caucasian (*n* = 6) and South Asian (*n* = 6). Different photographs of the target were used at encoding and retrieval to maintain reliably, generalizability, and to test face recognition rather than image recognition (31, 32).

#### Lineups.

**Fig. 2.**
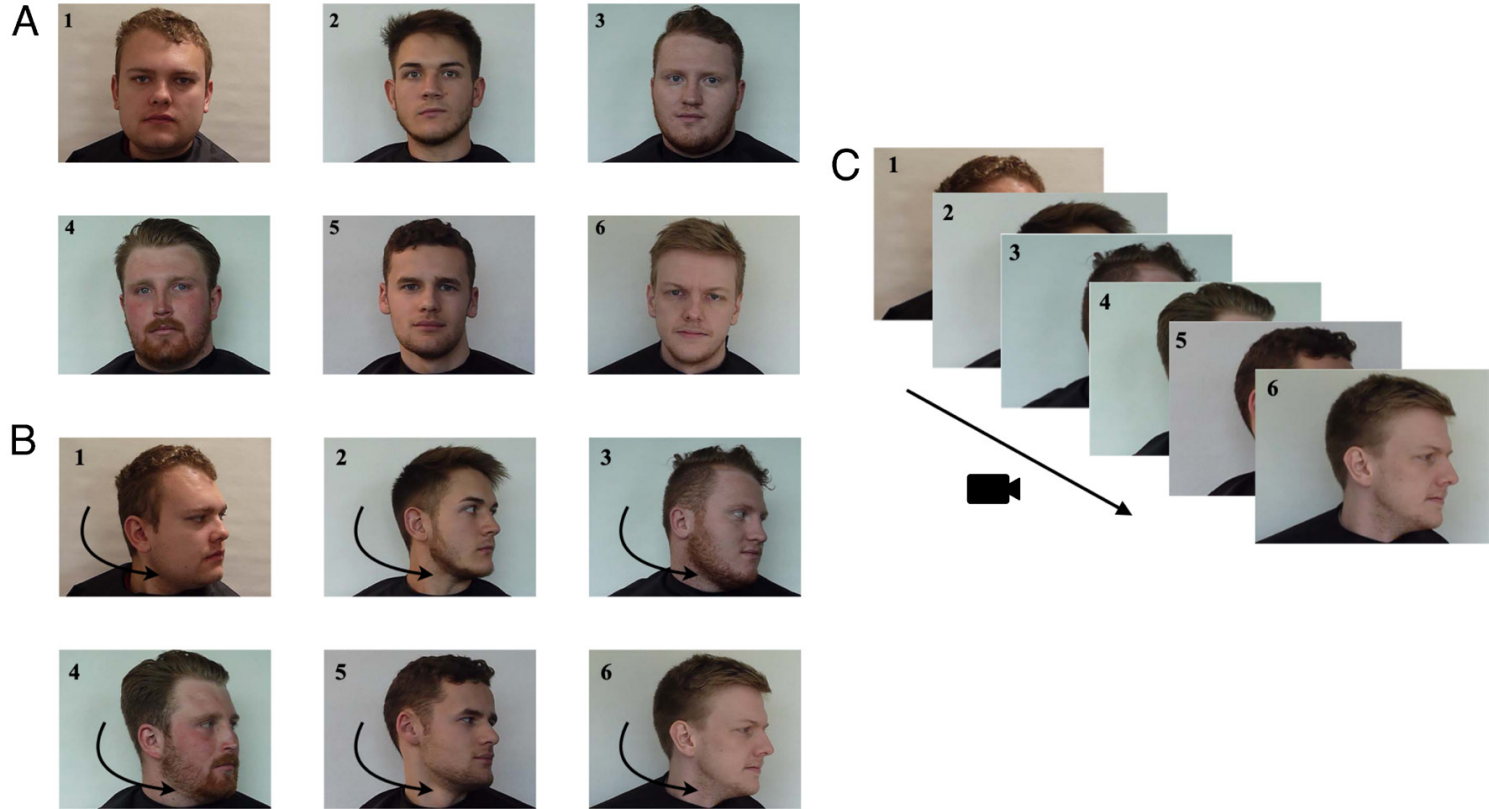
Visualization of the three lineup procedures. Note. Illustrations of the (*A*) simultaneous photo lineup, (*B*) simultaneous interactive lineup, and (*C*) sequential video lineup. In *A*, six faces were presented simultaneously in frontal pose. In *B*, six faces were presented simultaneously in frontal pose, and participants used the computer mouse to click on one face and rotate it, then all faces moved jointly together. In *C*, only one face was presented at a time via a 20-s video in which the face moved fluidly from front to profile, to the opposite profile, and back to the front. Adapted from ref. 20.

A six-person target-present lineup (containing the target and five fillers) and a six-person target-absent lineup (which does not contain the target face, but six fillers) for each target face was constructed for each lineup procedure (interactive, photo, video). Fillers were selected to match the targets’ physical appearances (e.g., sex, ethnicity). To minimize position effects, two lineups with randomised face orders were generated for each target. Colloff et al. (20) ensured that all images were taken with the same camera, background, and focal distance. Each member wore a cape to cover clothing and had no distinctive features (e.g., no jewellery, make-up, etc.). Mock-witness tests indicated the lineups were fair (20). See Fig. 2 for examples.

### Procedure.

Participants performed the experiment using a computer and were randomly assigned to a condition. First, consent and demographic information was collected. Next, participants were sequentially presented with twelve faces for 5 s each and were instructed to pay attention as they would be asked questions about the faces later. Participants then watched a 1-min cartoon about a cat and a mouse as a distractor task. Afterward, participants were presented with the instructions and told that they should attempt to identify the targets that they previously saw. Participants were informed that the target may not be present in the lineup, and they should reject the lineup if need be by answering “not present”. Participants in the interactive condition were instructed to use their computer mouse to click on one face and to drag it to see it from different angles. They were informed that all faces would move together and that it is crucial to engage with the faces as their mouse movements will be recorded. They could only proceed to the lineup decision once they had moved their mouse. Participants in the photo condition were presented with six frontal-pose faces simultaneously, and they could proceed after 30 s. Participants in the video condition were presented with six 20-s videos that were shown sequentially, and they could only proceed once all videos had been displayed.

Subsequently, the twelve lineups were presented in a random order. After each lineup, participants made a decision and provided their using an 11-point Likert-type rating scale, ranging from 0% “guessing” to 100% “completely certain.”

Finally, participants answered an attention check question about what happened in the distractor task video and reported whether they had experienced any technical difficulties. Participants were thanked and debriefed.

## Supplementary Material

Appendix 01 (PDF)

## Data Availability

csv data have been deposited in OSF (https://osf.io/rkafh/) (33).
