# Supplementary material for: Enabling witnesses to actively explore faces and reinstate study-test pose during a lineup increases discriminability
Source: Proc Natl Acad Sci U S A. 2023 Oct 2;120(41):e2301845120. doi: 10.1073/pnas.2301845120 (PMC10576112; doi:10.1073/pnas.2301845120)
Supplement: Supplementary file 1 — Appendix 01 (PDF) [file pnas.2301845120.sapp.pdf]

PNAS

Supporting Information for

Enabling witnesses to actively explore faces and reinstate study-test pose during a lineup increases discrimination accuracy

Marlene Meyer[1], Melissa F. Colloff[2], Tia C. Bennett[2], Edward Hirata[3], Amelia Kohl[2], Laura M. Stevens[2], Harriet M. J. Smith[4], Tobias Staudigl[5], and Heather D. Flowe[2]*

[1] Department of Sport and Health Sciences, Technical University of Munich, Germany.
[2] School of Psychology, University of Birmingham, United Kingdom.
[3] School of Psychology, University of Bath, United Kingdom.
[4] School of Psychology, Nottingham Trent University, United Kingdom.
[5] Department of Psychology, Ludwig-Maximilians-Universität München, Munich, Germany.
*Corresponding author – Heather D. Flowe
Email: h.flowe@bham.ac.uk

This PDF file includes:

Supporting text
Figures S1
Tables S1 to S5

Results: Decision Time and Viewpoint Analyses Across Lineup Conditions

(Non-Interactors removed)

Descriptive statistics for mean decision time (ms) by lineup and encoding conditions with non-interactors removed are displayed in Table S1. To determine whether participants in the interactive condition showed superior discriminability due to a longer exposure time at test, we compared how time spent viewing the lineups before making a decision varied between the three conditions. Welch's F-test showed that viewing time differed significantly between the three groups when the face was encoded in the frontal position, $F(2,2221) = 27.5$, $p <.001$, with those in the video condition ($M = 7971$, $SD = 24928$) viewing the faces significantly longer than those in the static condition ($M = 3545$, $SD = 6956$), $t(1315) = -5.87$, $p = <.001$, and the interactive condition ($M = 2592$, $SD = 7313$), $t(1339) = -7.10$, p <.001. Those in the static condition viewed the lineups for significantly longer than those in the interactive condition, $t(2681) = -3.45$, $p = .002$. The same was found for participants who encoded the stimuli in profile view, $F(2,2190) = 69.1$, $p < .001$, with those in the video condition ($M = 8265$, $SD = 18046$) viewing the faces significantly longer than those in the static condition (M =3697, SD = 7069), $t(1483) = -8.16$, $p < .001$, and the interactive condition ($M = 2339$, $SD = 4976$), $t(1325) = -10.92$, $p < .001$. Again, the static condition viewed the lineup for significantly longer than the interactive condition, $t(2449) = -5.83$, p < 001. Thus, we conclude that the superior discriminability displayed in the interactive condition is not due to an increased exposure to the stimuli at test.

Previous studies have shown evidence of pose reinstatement by participants in the interactive condition (evidenced by participants spending more time viewing the lineup faces in the same angle that they were encoded; e.g., Colloff et al., 2022). In our current study, the interactive lineup condition tracked participants' mouse movement coordinates, which we then drew on to calculate the total distance that the cursor moved per trial (using the 'mousetrap' package in Rstudio; Kieslich, 2022). We assumed that a larger distance traveled was reflective of increased interaction, which would be expected in the profile condition if participants were engaging in pose reinstatement (c.f., the front encoding condition, where interactivity would not be required for pose reinstatement). An independent samples Welch's t-test showed no significant difference between the profile (M = 5.59, SD = 6.11) and front (M = 5.50, SD = 3.54) conditions, $t(992) = 0.33$, $p = 0.740$, $d = 0.02$. It's possible that this is because all participants in the interactive condition were instructed to interact with the lineup, therefore increasing the motivation for participants in the front encoding condition to engage with the stimuli.

Testing Correlational and Distributional Accounts:

Maximum Likelihood Signal Detection Model Fits (Non-Interactors removed)

We fit another model allowing μ_{target} (d, the distance between the guilty and innocent distributions) and σ_b (the correlation) to differ across the lineup conditions, constraining the model-estimated σ_{target} to be the same across the lineup conditions, and allowing the confidence criteria to vary (see Table 4 in the main paper). We then fit a model constraining both d and the correlation to be the same across the lineup conditions

(model fits, front encoding: χ^2 (15) = 19.74, p = .182; profile encoding: χ^2 (15) = 36.95, p = .001). For front encoding, compared to this constrained model, allowing d to differ across the lineup conditions significantly improved the fit, χ^2 (2) = 7.81, p = .020, but allowing the correlation to differ across the lineup conditions did not, χ^2 (2) = 1.80, p = .407. For profile encoding, allowing d (χ^2 (2) = 24.63, $p < .001$) and the correlation (χ^2 (2) = 11.62, p = .003) to differ across the lineup conditions both significantly improved the fit, but the d effect was larger. Therefore, improved ability to discriminate innocent from guilty suspects in interactive compared to photo and video lineups appears to be predominantly due to the innocent and guilty memory strength distributions moving apart.

ROC and Modelling Results: All Subjects Including Non-Interactors

Lineup Identification Decisions

The number of target, filler and "Not Present" (reject) identification decisions (IDs) given to front- and profile-encoding conditions in simultaneous interactive, simultaneous photo and sequential video lineups are displayed in Table S3, with frequencies shown for different confidence ratings. As shown in Table S3, interactive lineups seem to yield enhanced performance for the profile-encoding condition compared to photo and video lineups, but there seem to be little difference in performance between lineup conditions for the front-encoding condition.

ROC Analysis

Figure S1 shows the partial ROC curves (A, C) and displays the complementary *p*AUC values (B, D) of the three lineup procedures (simultaneous interactive, simultaneous photo, and sequential video lineups).

To test if any differences were statistically significant, the *p*AUC was calculated. A specificity of .48 was used for the front-encoding condition analysis. There were no significant difference between the *p*AUC for the interactive condition (.156) and the *p*AUC for the photo condition (.145), $D = 0.734$, $p = .46$. Contrary to the predictions of the diagnostic-feature-detection theory, the *p*AUC for the interactive condition (.156) did not significantly differ from the *p*AUC for the video condition (.137), $D = 1.227$, $p = .11$ (one-tailed). Additionally, there were no significant differences between the *p*AUC for the photo condition (.145) and the *p*AUC for the video condition (.137), $D = .544$, $p = .29$ (one-tailed).

For the profile-encoding condition analysis, a specificity of .46 was used. In line with the hypotheses, the *p*AUC for the interactive condition (.131) was significantly greater than the *p*AUC for both the photo condition (.082), $D = 4.143$, $p < .001$ (one-tailed) and the *p*AUC for the video condition (.089), $D = 4.143$, $p < .001$ (one-tailed). In sum, for any false identification rate, interactive lineups enhanced the correct identification rate of target faces by 60% compared to photo lineups and by 47% compared to video lineups. Contrary to the hypothesis, there was no significant difference between the *p*AUC (.082) for the photo condition (.082) and the *p*AUC for the video condition (.089), $D = .569$, $p = .57$.

We used the same model-fitting procedure as outlined in the main paper. For front encoding (see Table S4) the *full model* explains the data well (χ^2 (14) = 12.83, p = .540); descriptively speaking, discriminability is similar across the three lineup conditions, interactive (d = 1.14), photo (d = 1.11), and video (d = 0.99). There were no significant differences in discriminability between interactive and photo lineups (χ^2 (1) = 0.07, p = .791), interactive and video lineups (χ^2 (1) = 1.61, p = .204), or photo and video lineups (χ^2 (1) = 1.05, p = .306).

For profile-encoding (see Table S5), the *full model* explains the data well (χ^2 (14) = 11.33, p = .660). Discriminability was significantly larger in interactive (d = 0.97) compared to photo (d = 0.60) lineups (χ^2 (1) = 14.22, $p < .001$), and interactive compared to video (d = 0.60) lineups, χ^2 (1) = 12.46, $p < .001$. There was no significant difference in discriminability between photo and video lineups, χ^2 (1) = 0.00, p = 1.000. Taken together, the results from Signal-Detection Model fit are consistent with the findings from ROC analysis for all subjects.

Figure S1.

pROC curves and pAUC statistics for all subjects (including non-interactors)

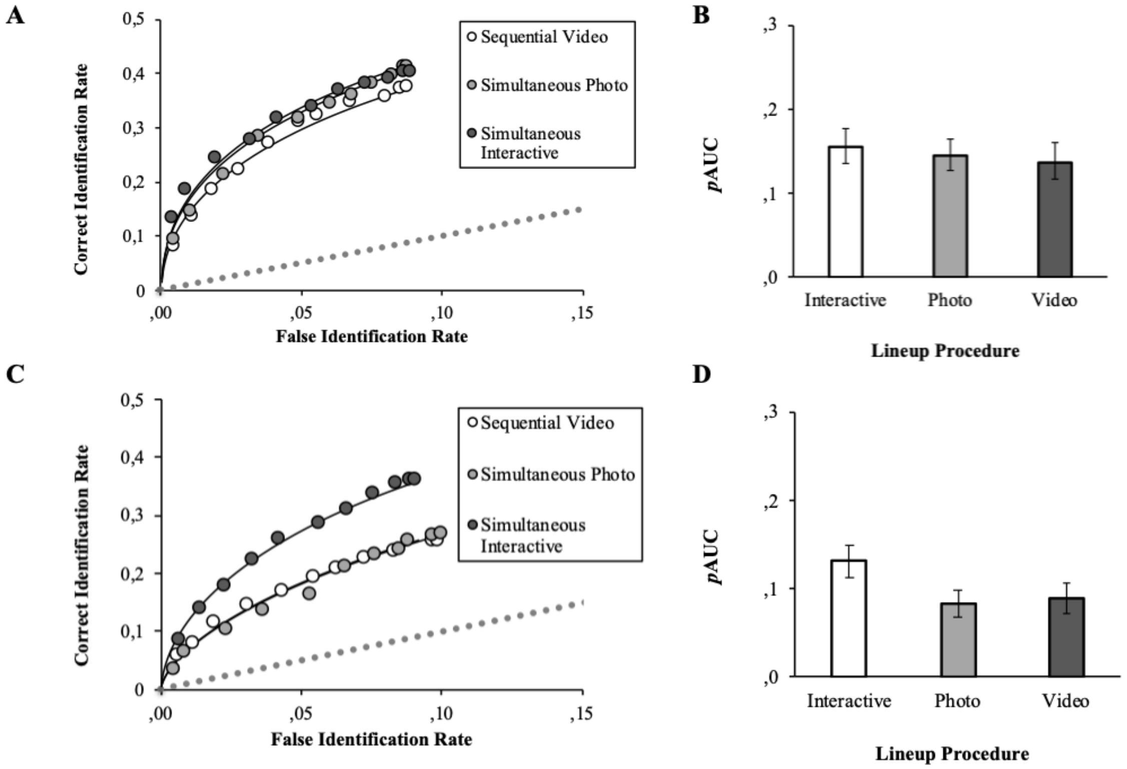

Note. *p*ROC curves and *p*AUC statistics for simultaneous interactive, simultaneous photo and sequential video lineups, separated for front- (A, B) and profile- (C, D) encoding conditions. ROC lines of best fit were plotted from numbers estimated by unequal-variance signal-detection models, see section Maximum Likelihood Signal-Detection Model Fits. Chance-level performance is indicated by dashed lines. For *p*AUC values (B, D), error lines are 95% CIs.

Table S1.

Descriptive Statistics for Decision Time Across the Interactive, Photo and Video Lineup Conditions (excluding non-interactive participants).

	**N**	**M**	***SD***	***SE***	***95% CI***
Profile-encoding					
Interactive	1404	2339	4976	133	[2079, 2599]
Photo	1368	3697	7069	191	[3322, 4072]
Video	1176	8265	18046	526	[7234, 9296]
Front-encoding					
Interactive	1320	2592	7313	201	[2197, 2986]
Photo	1392	3545	6956	186	[3180, 3911]
Video	1164	7971	24928	731	[6539, 9403]

Table S2.

Frequencies of Target, Filler, and Reject Identification Decisions Given with Different Confidence Ratings to Front- and Profile-Encoding Conditions in Interactive, Photo and Video Lineups. Excluding 31 Subjects from the Interactive Condition who Did Not Interact.

Confidence rating	Front-encoding					Profile-encoding				
	Target-present			Target-absent		Target-present			Target-absent	
	Target	Filler	Reject	Filler	Reject	Target	Filler	Reject	Filler	Reject
					Interactive					
0	0	7	2	8	2	0	2	6	5	7
10	6	8	6	13	6	3	9	5	14	7
20	4	11	7	22	10	7	15	11	23	10
30	6	8	15	26	17	11	15	18	25	27
40	13	16	9	21	17	10	25	14	19	25
50	7	16	16	30	44	15	18	16	44	23
60	20	9	16	20	30	17	20	11	24	21
70	16	10	7	22	22	21	15	19	25	30
80	25	5	11	15	13	18	16	4	21	21
90	21	7	11	9	11	29	16	9	21	9
100	55	5	9	7	19	44	14	15	18	49

Total	***173***	***102***	***109***	***193***	***191***	***175***	***165***	***128***	***239***	***229***
Proportion	***0.45***	***0.27***	***0.28***	***0.50***	***0.50***	***0.37***	***0.35***	***0.28***	***0.51***	***0.49***
**Photo**										
0	0	1	8	4	6	2	10	19	8	18
10	7	9	6	11	13	5	14	13	28	21
20	8	8	7	21	9	7	14	14	10	10
30	11	20	12	22	21	5	20	8	27	14
40	7	25	13	22	12	11	14	10	33	16
50	13	17	20	33	35	24	30	12	37	23
60	17	17	16	43	23	14	25	16	53	26
70	35	18	21	36	37	18	31	22	40	20
80	33	20	11	35	22	20	24	17	47	20
90	25	7	12	18	22	15	10	12	10	11
100	47	7	14	13	34	18	19	23	15	29
Total	***203***	***149***	***140***	***258***	***234***	***139***	***211***	***166***	***308***	***208***
Proportion	***0.41***	***0.30***	***0.29***	***0.48***	***0.52***	***0.27***	***0.40***	***0.33***	***0.60***	***0.40***
**Video**										
0	2	2	0	5	0	0	2	5	4	10
10	6	7	2	13	3	8	10	13	35	9

20	3	10	9	28	3	5	25	10	26	12
30	9	15	4	27	7	7	16	13	25	9
40	5	13	9	16	8	7	13	9	21	16
50	15	13	11	24	27	10	26	23	29	22
60	19	19	10	24	23	10	19	13	31	14
70	15	14	23	23	25	12	14	13	30	25
80	18	9	15	16	23	15	14	18	18	16
90	22	6	8	15	34	9	10	20	15	16
100	31	14	26	10	30	25	11	15	14	23
Total	***145***	***122***	***117***	***201***	***183***	***108***	***160***	***152***	***248***	***172***
Proportion	***0.38***	***0.32***	***0.30***	***0.52***	***0.48***	***0.26***	***0.38***	***0.36***	***0.59***	***0.41***

Note. Confidence ratings were collected as a percentage. Total rows contain the frequency of every identification decision collapsed over subjects and confidence. Proportion rows are calculated by dividing the amount of identification decisions by the number of lineups in a particular condition. For instance, the proportion of front-encoding target identifications in interactive lineups is computed by dividing the number of target identifications in target-present front-encoding interactive lineups by the total amount of target-present front-encoding interactive lineups, 173 / (173+102+109).

Table S3.

Frequencies of Target, Filler, and Reject Identification Decisions Given with Different Confidence Ratings to Front- and Profile-Encoding Conditions in Interactive, Photo and Video Lineups for all subjects (including non-interactors).

Confidence rating	Front-encoding					Profile-encoding				
	Target-present			Target-absent		Target-present			Target-absent	
	Target	Filler	Reject	Filler	Reject	Target	Filler	Reject	Filler	Reject
**Interactive**										
0	0	7	2	8	2	0	6	6	7	9
10	6	8	6	15	7	3	12	5	17	7
20	5	12	9	25	12	9	19	11	26	10
30	6	9	15	27	17	16	20	19	30	27
40	15	18	11	28	18	12	27	14	32	28
50	10	22	20	37	47	15	21	20	47	26
60	20	14	17	29	33	20	22	15	32	21
70	16	13	10	37	23	25	25	22	31	35
80	29	20	17	30	16	20	19	5	29	21
90	26	10	17	14	20	30	16	9	26	9
100	66	10	26	12	35	47	15	21	20	56
Total	***199***	***143***	***150***	***262***	***230***	***197***	***202***	***147***	***297***	***249***
Proportion	***0.40***	***0.29***	***0.31***	***0.53***	***0.47***	***0.36***	***0.37***	***0.27***	***0.54***	***0.46***
**Photo**										

0	0	1	8	4	6	2	10	19	8	18
10	7	9	6	11	13	5	14	13	28	21
20	8	8	7	21	9	7	14	14	10	10
30	11	20	12	22	21	5	20	8	27	14
40	7	25	13	22	12	11	14	10	33	16
50	13	17	20	33	35	24	30	12	37	23
60	17	17	16	43	23	14	25	16	53	26
70	35	18	21	36	37	18	31	22	40	20
80	33	20	11	35	22	20	24	17	47	20
90	25	7	12	18	22	15	10	12	10	11
100	47	7	14	13	34	18	19	23	15	29
Total	***203***	***149***	***140***	***258***	***234***	***139***	***211***	***166***	***308***	***208***
Proportion	***0.41***	***0.30***	***0.29***	***0.52***	***0.48***	***0.27***	***0.40***	***0.33***	***0.60***	***0.40***
**Video**										
0	2	2	0	5	0	0	2	5	4	10
10	6	7	2	13	3	8	10	13	35	9
20	3	10	9	28	3	5	25	10	26	12
30	9	15	4	27	7	7	16	13	25	9
40	5	13	9	16	8	7	13	9	21	16
50	15	13	11	24	27	10	26	23	29	22
60	19	19	10	24	23	10	19	13	31	14
70	15	14	23	23	25	12	14	13	30	25
80	18	9	15	16	23	15	14	18	18	16
90	22	6	8	15	34	9	10	20	15	16
100	31	14	26	10	30	25	11	15	14	23

Total	***145***	***122***	***117***	***201***	***183***	***108***	***160***	***152***	***248***	***172***
Proportion	***0.38***	***0.32***	***0.30***	***0.52***	***0.48***	***0.26***	***0.38***	***0.36***	***0.59***	***0.41***

Note. Confidence ratings were collected as a percentage. Total rows contain the frequency of every identification decision collapsed over subjects and confidence. Proportion rows are calculated by dividing the amount of identification decisions by the number of lineups in a particular condition. For instance, the proportion of front-encoding target identifications in interactive lineups is computed by dividing the number of target identifications in target-present front-encoding interactive lineups by the total amount of target-present front-encoding interactive lineups, 199 / (199+143+150).

Table S4.

Fitted Models for the Interactive, Photo and Video Lineup d Comparisons for Front-Encoding for All Subjects (Including Non-interactors)

**Model**	μ_{target} / ***d***	σ_{target}	c_1	c_2	c_3
Full					
Interactive	1.14		1.21	1.84	2.32
Photo	1.11	1.27	1.20	1.82	2.40
Video	0.99		1.20	1.90	2.30
Model-fit		χ^2 (14) = 12.83, p = .540			
Reduced Interactive-Photo Comparison					
Interactive	**1.13**		1.21	1.84	2.31
Photo		1.27	1.20	1.82	2.40
Video	**0.99**		1.20	1.90	2.30
Model-fit		χ^2 (15) = 12.90, p = .610			
Reduced Interactive-Video Comparison					
Interactive	**1.08**		1.20	1.84	2.30
Photo	1.11	1.27	1.20	1.82	2.40
Video	**1.08**		1.21	1.91	2.32
Model-fit		χ^2 (15) = 14.44, p = .492			
Reduced Photo-Video Comparison					
Interactive	1.14		1.21	1.84	2.32
Photo	1.60	1.27	1.19	1.81	2.39
Video			1.20	1.91	2.32
Model-fit		χ^2 (15) = 13.88, p = .535			

Note. In the *full model*, *d* varies across conditions. In the *reduced models*, the two procedures that are compared are restricted to an equal *d*. In both full and reduced models, model-estimated σ_{target} was constrained to be the same over conditions, and c_1, c_2 and c_3 were free to vary. Model-fit rows represent the goodness-of-fit statistic.

Table S5.

Fitted Models for the Interactive, Photo and Video Lineup Comparisons for Profile-Encoding for All Subjects (Including Non-interactors)

Model	μ_{target} / d	σ_{target}	c_1	c_2	c_3
Full					
Interactive	0.97		1.14	1.81	2.20
Photo	0.60	1.17	1.09	1.77	2.34
Video	0.60		1.13	1.86	2.27
Model-fit		χ^2 (14) = 11.33, p = .660			
Reduced Interactive-Photo Comparison					
Interactive	0.79		1.12	1.79	2.18
Photo		1.18	1.11	1.79	2.37
Video	0.59		1.12	1.86	2.27
Model-fit		χ^2 (15) = 25.55, p = .043			
Reduced Interactive-Video Comparison					
Interactive	0.81		1.13	1.79	2.18
Photo	0.59	1.17	1.09	1.77	2.34
Video	0.81		1.15	1.89	2.30
Model-fit		χ^2 (15) = 23.79, p = .069			
Reduced Photo-Video Comparison					
Interactive	0.97		1.14	1.81	2.20
Photo	0.60	1.17	1.09	1.77	2.34
Video			1.13	1.86	2.27
Model-fit		χ^2 (15) = 11.33, p = .729			

Note. In the *full model*, d varies across conditions. In the *reduced models*, the two procedures that are compared are restricted to an equal d. In both full and reduced models, model-estimated σ_{target} was constrained to be the same over conditions, and c_1, c_2 and c_3 were free to vary. Model-fit rows represent the goodness-of-fit statistic.
